# Product and Process Fingerprint for Nanosecond Pulsed Laser Ablated Superhydrophobic Surface

**DOI:** 10.3390/mi10030177

**Published:** 2019-03-07

**Authors:** Yukui Cai, Xichun Luo, Zhanqiang Liu, Yi Qin, Wenlong Chang, Yazhou Sun

**Affiliations:** 1 Centre for Precision Manufacturing, DMEM, University of Strathclyde, Glasgow G1 1XJ, UK; yukui.cai@strath.ac.uk (Y.C.); qin.yi@strath.ac.uk (Y.Q.); wenlong.chang@strath.ac.uk (W.C.); 2 School of Mechanical Engineering, Shandong University, Jinan 250061, China; melius@sdu.edu.cn; 3 Key Laboratory of High Efficiency and Clean Mechanical Manufacture of MOE/Key National, Demonstration Center for Experimental Mechanical Engineering Education, Jinan 250061, China; 4 School of Mechatronics Engineering, Harbin Institute of Technology, Harbin 150001, China; sunyzh@hit.edu.cn

**Keywords:** laser ablation, superhydrophobic surface, process fingerprint, product fingerprint, surface morphology

## Abstract

Superhydrophobic surfaces have attracted extensive attention over the last few decades. It is mainly due to their capabilities of providing several interesting functions, such as self-cleaning, corrosion resistance, anti-icing and drag reduction. Nanosecond pulsed laser ablation is considered as a promising technique to fabricate superhydrophobic structures. Many pieces of research have proved that machined surface morphology has a significant effect on the hydrophobicity of a specimen. However, few quantitative investigations were conducted to identify effective process parameters and surface characterization parameters for laser-ablated microstructures which are sensitive to the hydrophobicity of the microstructured surface. This paper proposed and reveals for the first time, the concepts of process and product fingerprints for laser ablated superhydrophobic surface through experimental investigation and statistical analysis. The results of correlation analysis showed that a newly proposed dimensionless functional parameter in this paper, R_hy_, i.e., the average ratio of Rz to Rsm is the most sensitive surface characterization parameter to the water contact angle of the specimen, which can be regarded as the product fingerprint. It also proposes another new process parameter, average laser pulse energy per unit area of the specimen (*I_s_*), as the best process fingerprint which can be used to control the product fingerprint R_hy_. The threshold value of R_hy_ and *I_s_* are 0.41 and 536 J/mm^2^ respectively, which help to ensure the superhydrophobicity (contact angle larger than 150°) of the specimen in the laser ablation process. Therefore, the process and product fingerprints overcome the research challenge of the so-called inverse problem in manufacturing as they can be used to determine the required process parameters and surface topography according to the specification of superhydrophobicity.

## 1. Introduction

Superhydrophobic surfaces are defined as those having a water contact angle larger than 150° and sliding angle less than 10°. Artificial superhydrophobic surfaces, created by surface structuring or coating, have received tremendous attention in recent years. It is mainly due to their capabilities of providing several interesting functions, such as self-cleaning, corrosion resistance, anti-icing and drag reduction [1,2,3,4,5,6]. Surface chemical composition and morphology are two critical factors in determining their hydrophobicity [7,8,9]. The surface chemical composition affects the intrinsic contact angle, which can be measured by a liquid droplet deposited on a smooth surface. However, in artificial or natural materials, the maximum intrinsic contact angle is only approximately 120° [8,9]. For this reason, more and more structuring technologies have been developed for the fabrication of superhydrophobic surfaces, including wet chemical reaction, lithography, rolling, 3D printing, micro milling and laser ablation [2,6,10,11,12,13,14,15] etc. 

Recently, laser ablation process has been demonstrated as a promising technique to fabricate superhydrophobic structures on varied materials, such as copper, aluminium, steel and glass [15,16,17,18,19,20,21,22,23,24]. Yang et al. investigated the wettability transition mechanism of laser ablated aluminium substrate, the results indicated that laser-ablated microstructures had the amplified effects on the hydrophobicity of the specimen [24]. Long et al. reported the effect of the laser pulse energy and width on the morphology of micro/nanostructures on a copper surface. They found that the morphology of the laser ablated structures is more sensitive to the laser pulse energy when nanosecond lasers with long pulse widths are used. Slightly decreasing the laser pulse energy results in the formation of no hierarchical micro- and nanostructures [17,20]. Gregorcic et al. fabricated a 316L stainless steel specimen with a pitch of 50 μm at average pulse power of 0.6 W and 97% pulse overlapping rate and achieved a static contact angle of 153° [18]. Long and Gregorcic both reported that variation of the pitch of channels resulted in completely different surface morphologies—from the highly porous surface to well-separated microchannels, which width and depth depend on laser fluence [18]. Duong Ta et al. concluded that surface roughness could be well controlled by laser power. The arithmetical mean height, Sa increased linearly when laser fluence was higher than 33 J/cm^2^. The roughness was around 2 and 7 times larger than that of the untextured surface under fluences of 36 and 48 J/cm^2^, respectively [23]. In addition, the effect of laser fluence and line separation on the contact angle of laser structured surfaces were investigated. Experimental results showed that the specimens possess superhydrophobicity has pitches of 50–150 μm and machined at the laser fluence of 36 J/cm^2^ [23]. M. Conradi discovered that higher line density resulted in a higher contact angle. However, the average surface roughness Sa increased first then further decreased gradually with the increase of line density [19]. Thus, these researches have indicated that the laser machining parameters would significantly influence the hydrophobicity of the specimens while surface topography is a crucial factor to determine the superhydrophobicity of the specimen. However, there has been little systematic research exploring the correlation between surface topography and hydrophobicity of the specimen. Furtherly, the second challenge is to find out the most effective process parameter and surface characterization parameter for these microstructures which are sensitive to the hydrophobicity of the microstructured surface.

The identification of “product and process fingerprints” of laser ablated surface is a possible solution to solve the above issues. The concept of “product fingerprint” refers to those unique measurable characteristics (e.g., surface characterization parameters) on the laser ablated specimen that, if kept under control and within specifications, will ensure that the specimen possesses superhydrophobicity as required. The product fingerprint must be also sensitive to the variation of process parameters, hence it can be well-controlled by process parameters. For laser ablation process, since the surface characterization parameters are highly related to laser machining parameters, the “Process fingerprint” is defined as a specific process parameter to be controlled in order to maintain the manufacture of the specimen within the specified surface characterization parameters. The product and process fingerprints can be used as an objective function within an optimization tool to assist to determine the required surface topography and process parameters for the superhydrophobic surface.

The purpose of this paper is to reveal the product and process fingerprints for the laser ablation process of superhydrophobic surfaces on 316L stainless steel. A more generalized description can be achieved by linking laser machining parameters, surface characterization parameters and hydrophobicity of the specimen, which is beneficial to precise control of hydrophobicity and simultaneously enhancing its robustness. Therefore, product and process fingerprints are expected to provide a solution to the so-called inverse problem in manufacturing, which means the laser machining parameters and surface characterization parameters can be determined according to the required hydrophobicity, i.e., contact angle. Firstly, analysis of potential process and product fingerprint candidates will be carried out. Then, the most appropriate product fingerprint will be determined from values of Spearman and Kendall rank correlation coefficients according to the experimental results. Thirdly, a new process parameter will be put forward and chosen as the best process fingerprint. Lastly, the correlation between process fingerprint and functional performance, i.e., contact angle will be explored.

## 2. Analysis of Process and Product Fingerprints

**Figure 1:**
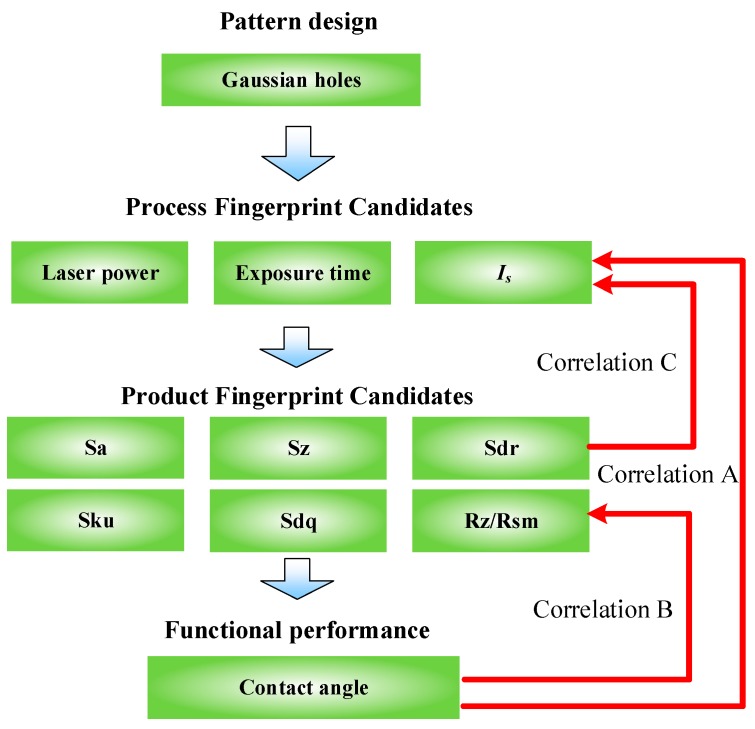
Concept of the process and product fingerprints in laser ablation of the superhydrophobic surface.

Figure 1 illustrates the concept of process and product fingerprints in the laser ablation process for obtaining the superhydrophobic surface with an array of Gaussian holes of designed geometry. The comparison of all the potential candidates of process and product fingerprints will be discussed in detail later. Most research performed to date has focused on the correlation A; i.e., the effect of laser machining parameters on the contact angle of specimens. However, correlation A is actually composed of correlation B and C. Correlation B refers to the relationship between contact angle and product fingerprint, which is used to explain the underlying mechanism of effect of surface topography on hydrophobicity. Correlation C can describe the relationship between the process fingerprint and product fingerprint, to explore how the process parameters affect the surface topography. Thus, product fingerprint is a bridge to connect process parameters and functional performance-contact angle.

### 2.1. Analysis of Process Fingerprint Candidates: Laser Power, Exposure Time, Laser Pulse Energy Per Unit Area of Specimen

#### 2.1.1. Laser Power (P)

In a nanosecond pulsed laser ablation process, the absorbed energy from the laser pulse melts the stainless steel and heats it to a temperature at which the atoms gain sufficient energy to enter into a gaseous state. Due to the vapour and plasma pressure, the molten materials are partially ejected from the cavity and form surface debris. At the end of a pulse, the heat quickly dissipates into the bulk of the work material and recast layer are formed. Therefore, laser power is a good candidate of process fingerprint as it determines the laser fluence which directly affects the formation of microstructures. The relationship between laser power, pulse repetition rate and peak power can be expressed as:

(1) $$E_{p}=\frac{P}{f_{p}}$$

(2) $$P_{peak}=\frac{E_{p}}{\Delta \tau }$$

where *P* is laser average power, *f*_p_ is pulse repetition rate, *E_p_* is the energy of a single pulse, *P*_peak_ is the peak power of laser and Δτ is the pulse duration, respectively.

#### 2.1.2. Exposure Time (t)

**Figure 2:**
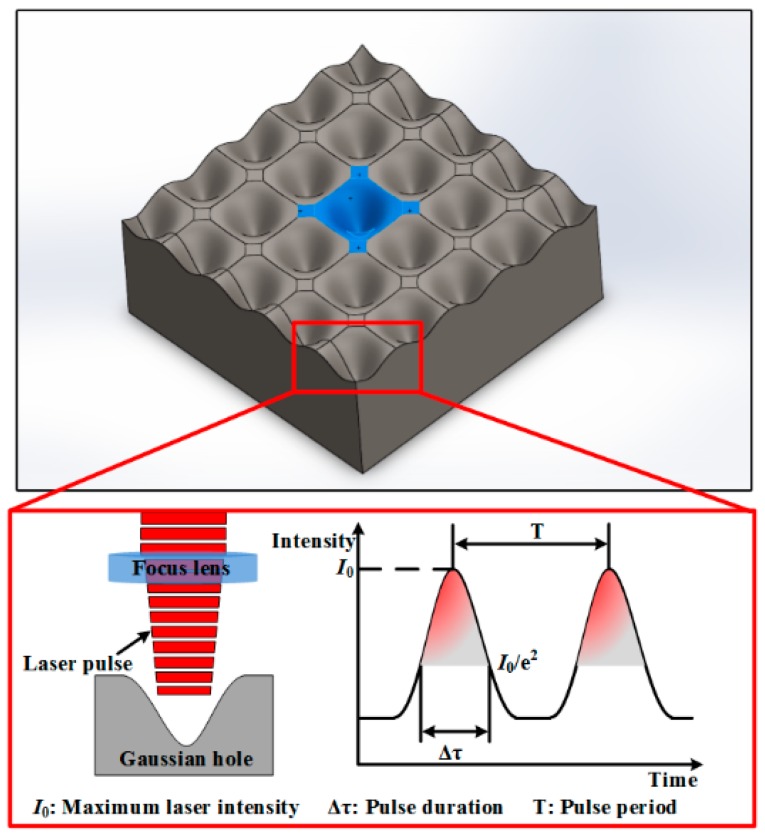
Schematic of periodic Gaussian holes machined by the laser ablation process.

For substrate with periodic Gaussian holes generated by the laser ablation process, the exposure time t means the machining time for a single Gaussian hole, which determines the number of laser pulses that irradiated the surface. It has a significant effect on the dimension and morphology of Gaussian holes. As shown in Figure 2, the relationship between the number of irradiated pulse N and exposure time t can be expressed as:

(3) $$N=\frac{t}{T}$$

where T is the pulse period.

Laser pulse energy per unit area of the specimen *I*_s_.

*I_s_* means the average laser pulse energy irradiated on a unit area of the specimen. This parameter depends on pulse repetition rate *f*_p_ and exposure time t. It can be expressed as:

(4) $$I_{s}=\frac{t*f_{p}*E_{p}{\left(\frac{L}{\mathrm{Pitch}}\right)}^{2}}{L^{2}}$$

According to Equation (1), $f_{p}*E_{p}=P$, hence Equation (4) can be simplified as:

(5) $$I_{s}=\frac{t*P}{{\mathrm{Pitch}}^{2}}$$

where pitch is the distance between adjacent Gaussian holes, and L is the length of the specimen.

### 2.2. Analysis of Product Fingerprint Candidates: Sa, Sz, Sku, Sdr, Sdq, R_hy_

In literature, two typical models have been developed to describe the behavior of a droplet on rough surfaces, i.e., the Wenzel and Cassie-Baxter models [25,26]. According to the Wenzel model, the droplet maintains contact with the structures and penetrates the asperities, and the surface contact area is increased. In addition, the contact angle *θ_w_* can be described as:

(6) $$\cos\theta_{w}=r\cos\theta$$

(7) $$r=\frac{\mathrm{actual}\;\mathrm{surface}\;\mathrm{area}}{\mathrm{planar}\;\mathrm{area}}$$

where, *r* is the roughness factor, which defined as the ratio of the actual area of the solid surface to the planar area. *θ* is the intrinsic contact angle of the material.

Alternatively, according to the Cassie-Baxter model, the droplet is not able to penetrate the microstructure spaces. However, in order to ensure the droplet cannot connect with the bottom of the microstructures, so the sag in height of water droplet between microstructures should be smaller than the depth of microstructures. Moreover, deep microstructures will help to form stable air pockets under the water droplet. Stable air pockets underneath the water droplet help the formation of superhydrophobicity with strong resistance against transition to the Wenzel state. Hence, sufficient depth of microstructure is essential to realize Cassie–Baxter state of the water droplet. The static contact angle *θ_CB_* can be expressed as:

(8) $$\cos\theta_{CB}=-1+f\left(1+\cos\theta \right)$$

(9) $$f=\frac{\mathrm{actual}\;\mathrm{solid}\;\mathrm{and}\;\mathrm{liquid}\;\mathrm{contact}\;\mathrm{area}}{\mathrm{planar}\;\mathrm{area}}$$

where *f* is the fraction of the solid-liquid contact area.

The above analysis proves that the contact angles obtained in both Wenzel and Cassie-Baxter states are highly related to the vertical and horizontal feature of surface topography. Six surface characterization parameters that most probably correlated with the hydrophobicity of specimens are listed in Table 1. Sa, Sz and Sku are roughness parameters to characterize the height of the surface. Sdr, Sdq, R_hy_ are hybrid parameters which determined from both height and horizontal parameters of the surface. For a rough surface, Sdr means the additional surface area contributed by the texture as compared to the planar definition area. Therefore, 1+Sdr has the same meaning as the roughness factor *r* in the Wenzel state. 

Theoretical analysis proved that microstructures should have a high aspect ratio to provide a larger surface area and a smaller separation distance which will help to improve the stabilization of the solid–liquid–air composite interface [27]. However, present functional parameters cannot reflect the aspect ratio of surface asperities. Hence, R_hy_ is proposed for the first time as a dimensionless functional parameter in this research and defined as the average ratio of Rz to Rsm. The subscript “hy” is the short abbreviation of hydrophobicity. The R_hy_ is calculated from the average value of 60 lines that evenly distributed on the structured surface horizontally and vertically. A surface with large R_hy_ can be obtained from a large Rz or smaller Rsm, which means the features of the surface should have a large depth or smaller separation distance (i.e., high density) in the horizontal direction.

## 3. Experimental Details

**Figure 3:**
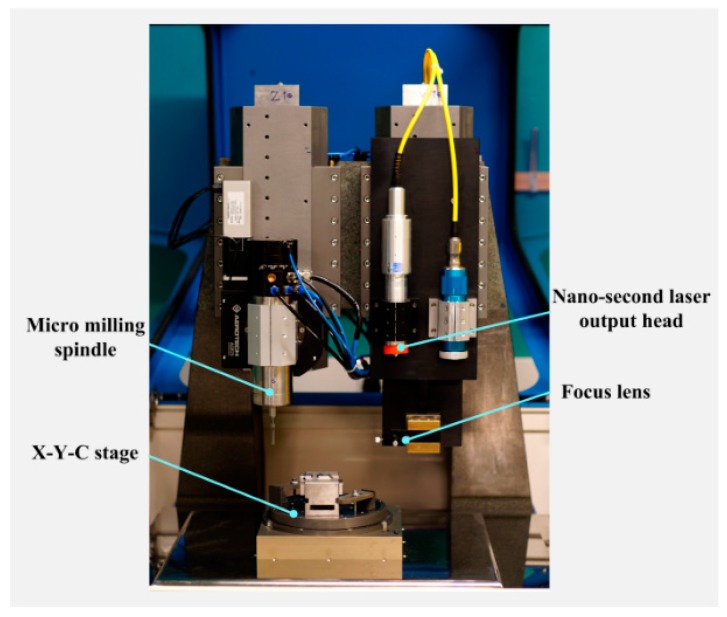
Experimental setup for laser machining trials.

Laser machining experiments were carried out on AISI 316L stainless steel by varying the process parameters in order to identify the best product and process fingerprints. All the experiments were carried out on a hybrid ultra-precision machine, as shown in Figure 3. It is equipped with a nanosecond pulsed fiber laser which has a central emission wavelength of 1064 nm. The laser source has a nominal average output power of 20 W and its maximum pulse repetition rate is 200 kHz. For a pulse repetition rate of 20 kHz, the average pulse duration is 100 ns and pulse energy is 1 mJ. The laser machining parameters are listed in Table 2 and Table 3. After the laser ablation process, the specimens were cleaned ultrasonically with deionized water, acetone and ethanol successively. Then the prepared specimens were silanized in a vacuum oven using silane reagent (1H, 1H, 2H, 2H-Perfluorooctyltriethoxysilane, 97%, Alfa Aesar Ltd., Ward Hill, MA, USA), at 100 °C for 12 h to reduce their surface free energies.

The surface topography and varied surface characterization parameters of the laser structured surface were measured by a 3D laser scanning confocal microscope (VK-250, Keyence Corporation, Osaka, Japan). The static contact angle on surfaces was measured by a drop shape analyzer (Kruss Ltd., Hamburg, Germany). The selected water droplet volume was 5 μL. For each specimen, the contact angle of the water droplet was measured three times and the average value was adopted.

## 4. Results and Discussion

### 4.1. Analysis of Product Fingerprint: Sa, Sz, Sku, Sdr, Sdq, R_hy_

The investigation of experimental results was carried out to identify the product fingerprint from six candidates related to surface topography. The product fingerprint is the indicator that has the highest level of correlation to contact angle. In this research, the Spearman rank correlation coefficient and Kendall rank correlation coefficient were employed to determine the product fingerprint. Spearman rank correlation coefficient evaluates how strong the correlation between two variables can be defined by a monotonic function. It measures the strength and direction of the monotonic association between two variables, a perfect Spearman correlation of +1 or −1 occurs when each variable is a perfect monotone function of the other [28]. A positive Spearman correlation coefficient corresponds to an increasing monotonic trend between two variables, while a negative value means a decreasing monotonic trend. In addition, Spearman rank correlation coefficient is appropriate for data that is not normally distributed. It can be used to identify a non-linear correlation between two variables. Kendall rank correlation coefficient is a statistic used to measure the ordinal association between two variables [29]. However, unlike the Spearman coefficient, Kendall rank correlation coefficient only considers directional agreement while does not consider the difference between ranks. Therefore, this coefficient is more appropriate for discrete data. This coefficient returns a value of −1 to 1, where 0 is no correlation, 1 is a perfect positive correlation, and −1 is a perfect negative correlation. In most cases, the interpretations of Spearman and Kendall rank correlation coefficients are very similar and thus invariably lead to the same inferences. The above two coefficients were combined to determine the product fingerprint that has the maximum absolute value. The strength of the correlation between the variables can be evaluated by the absolute value of coefficients, as shown in Table 4.

**Figure 4:**
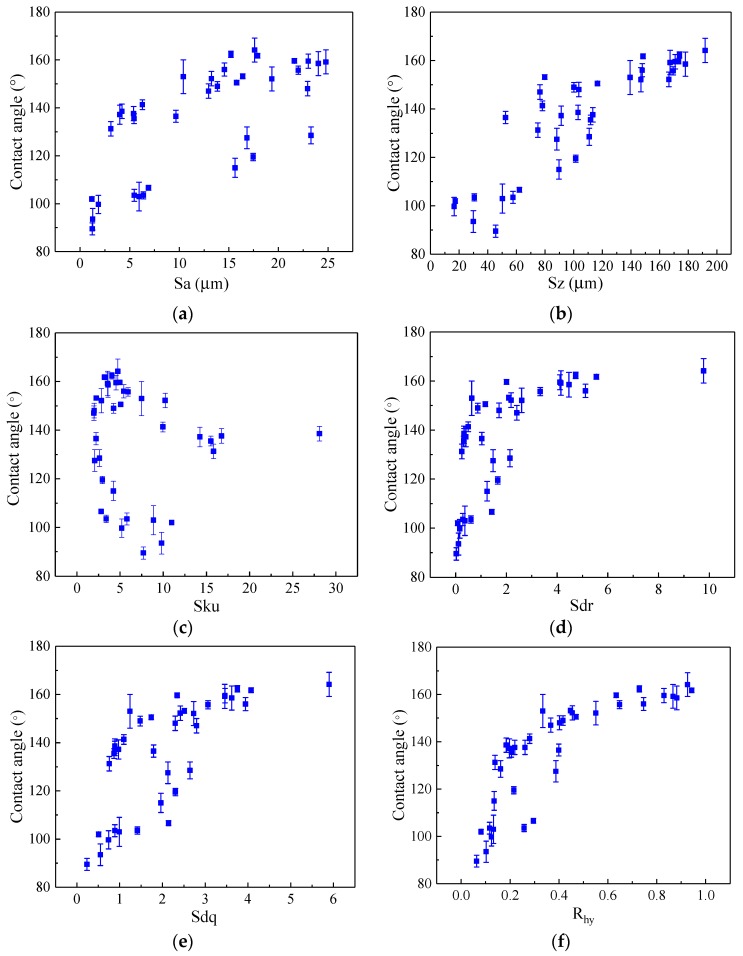
Influence of the product fingerprint candidates on the contact angle for Gaussian hole pattern: (**a**) Sa; (**b**) Sz; (**c**) Sku; (**d**) Sdr; (**e**) Sdq; (**f**) R_hy_.

Figure 4 shows scatter plots between the contact angle and the six candidates of product fingerprint. With the increase of Sa, Sz, Sdr, Sdq and R_hy_, the contact angle shows an increasing trend. It should be noted that a good linear relationship appears between Sz and contact angle, which is similar to the authors’ previous study [15]. However, it can be observed that there is no apparent correlation between Sku and contact angle (Figure 4c). As shown in Figure 4d, increasing Sdr from 0.02 to 4.1 leads to contact angle increase rapidly from 89.5° to 159°, but it has a minor impact on the contact angle when Sdr was further increased from 4.1 to 9.8. As Figure 4f indicates, the contact angle increases gradually from 89.5° to 164° with the value of R_hy_ increasing from 0.06 to 0.94. 

**Figure 5:**
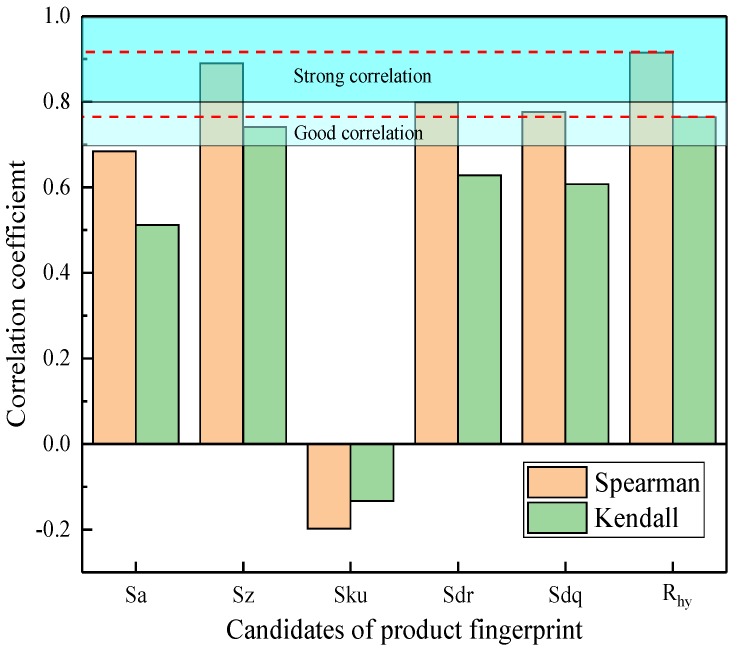
Spearman and Kendall rank correlation coefficient between the contact angle and six candidates of product fingerprint.

Figure 5 shows the variation of Spearman and Kendall rank correlation coefficient between contact angle and candidates of product fingerprint. According to the criterion in Table 4, Sz and R_hy_ both have larger Spearman rank correlation coefficients with the contact angle, which are 0.89 and 0.92 respectively. The Kendall rank correlation coefficient among Sz, R_hy_ and contact angle are 0.74 and 0.76. Thus, the results of Figure 5 suggest that R_hy_ should be determined as the best product fingerprint as it has the maximum Spearman and Kendall rank correlation coefficients.

According to the results in Figure 4f, an empirical equation was deduced to correlate the experimental R_hy_ and contact angle. The equation is expressed as:

(10) $$\theta_{A}=a-b{*e}^{c{*R}_{\mathrm{hy}}}$$

where, $\theta_{A}$ is contact angle; *a*, *b* and *c* are constant values, equal to 164, 105 and −4.9 respectively.

**Figure 6:**
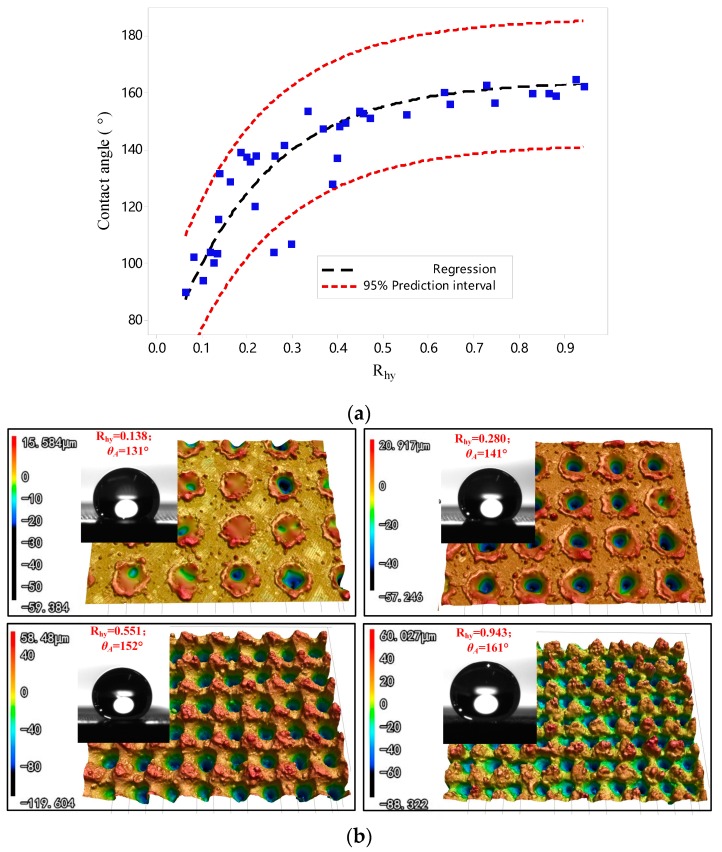
(**a**) Fitted line by exponential function between R_hy_ and contact angle; (**b**) Surface morphology and shape of water drops on specimens with a different value of R_hy_.

As shown in Figure 6a, the regression curve has good precision to simulate the experimental data. We found that coefficient “*a*” means the maximum contact angle (164° in this research), the value of “*b*” is equal to the initial contact angle (105°) of 316L stainless steel after chemical modification. Thus, the contact angle of the specimen is highly related to its maximum contact angle, initial contact angle on a smooth surface and hydrophobicity functional parameter R_hy_. According to Equation (10), the value of R_hy_ is 0.41 when $\theta_{A}$ = 150. Thus, 0.41 can be regarded as the threshold value of R_hy_ that ensure water contact angle of the specimen higher than 150°.

The dimensionless ratio R_hy_ is the most sensitive candidate parameter for contact angle of the specimen, which can therefore, be regarded as product fingerprint. In literature, many studies proved that a high density of microstructures and smaller period of microstructure will help decrease solid-liquid contact area and increase its hydrophobicity [22,30]. With the increase of R_hy_ from 0.138 to 0.943 (Figure 6b), Rsm decreased from 137.0 μm to 81.8 μm. Therefore, the density of peaks shows a significant increasing trend. Moreover, the depth of microstructures shows an increasing trend, due to average Rz increased from 18.9 μm to 77.2 μm. Therefore, it can be concluded that the superhydrophobicity will benefit from the increase of R_hy_.

### 4.2. Analysis of Process Fingerprints: P, t and I_s_

**Figure 7:**
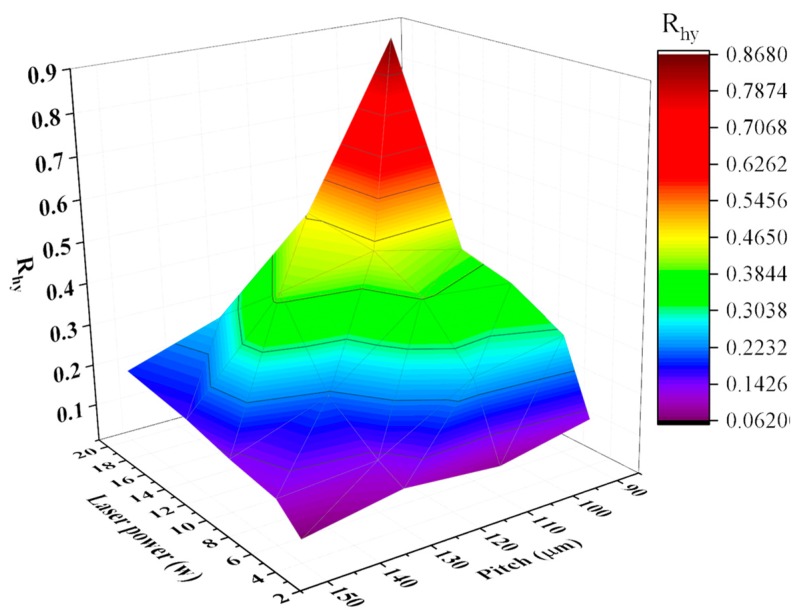
3D colormap of the product fingerprint (R_hy_) as a function of laser power and pitch of Gaussian hole.

The above section proves that R_hy_ is the most appropriate product fingerprint to the laser ablated superhydrophobic structures on 316L stainless steel. In this section, further analysis of the experimental results will be performed to identify the best process fingerprint from the candidates P, t and *I_s_*, i.e., the process fingerprint which has the strongest correlation with R_hy_. The control of process fingerprints helps to choose appropriate process parameter to obtain a surface with R_hy_ greater than the threshold value (R_hy_ > 0.41). The correlation among laser power, pitch of Gaussian hole and R_hy_ is shown in Figure 7. It shows that higher laser power and smaller pitch lead to a higher value of R_hy_. Laser power and pitch of structures have combined effects on the value of R_hy_.

**Figure 8:**
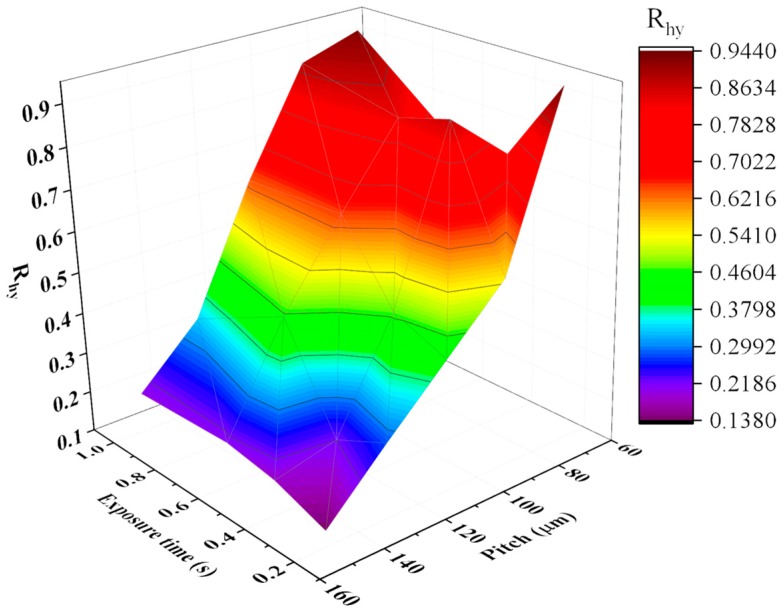
3D colormap of the product fingerprint (R_hy_) as a function of exposure time and pitch of Gaussian holes.

The effect of exposure time t and pitch of Gaussian holes on the value of R_hy_ is presented in Figure 8. There is no significant linear correlation between exposure time and R_hy_, but it does not mean exposure time has no effect on R_hy_. As a whole, it can be found that the value of R_hy_ shows a significant increasing trend with the reduction of pitch from 150 μm to 70 μm.

**Figure 9:**
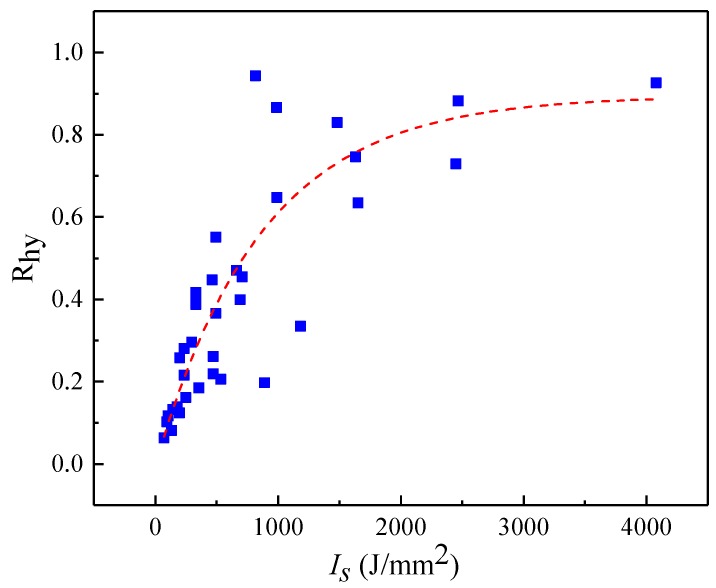
Scatter plots and fitted curve of R_hy_ and *I_s_*.

The above analysis shows that laser power, pitch and exposure time have a collective influence on R_hy_. Focusing one of them and ignoring the other two would lead to the determined correlation only effective in certain partial conditions. For instance, the R_hy_ will increase with laser power, but only valid at a precondition of constant pitch and exposure time. Therefore, a comprehensive factor *I*_s_ was designed to represent the combined influence of laser power, pitch and exposure time. *I_s_* means the energy intensity that irradiated on the unit area of the specimen and can be calculated by the Equation (5). *I_s_* is proportional to the laser power *P* and the exposure time t, but inversely proportional to the square of the pitch of the microstructures. Figure 9 reveals that the increasing *I_s_* leads R_hy_ increase rapidly at first, and then level off to become asymptotic to the upper limit. The presence of upper limit means the further increased laser power, exposure time and smaller pitch cannot lead to a further increase of R_hy_. The correlation between *I_s_* and R_hy_ can be expressed as Equation (11). According to the calculation result, *I_s_* should be greater than 536 J/mm^2^ to ensure R_hy_ greater than 0.41, hence the contact angle of the specimen will be larger than 150°.

(11) $$R_{\mathrm{hy}}=0.895-0.898*0.9985^{I_{s}}$$

Therefore, the increased *I_s_* leads to rapidly increase of R_hy_, the correlation between R_hy_ can be described by the exponential function. *I_s_* is the most sensitive parameters among the investigated three process fingerprint candidates, so it is the best process fingerprint that can be used to control surface morphology, especially the product fingerprint R_hy_.

### 4.3. Correlation Between Laser Machining Parameters and Contact Angle

**Figure 10:**
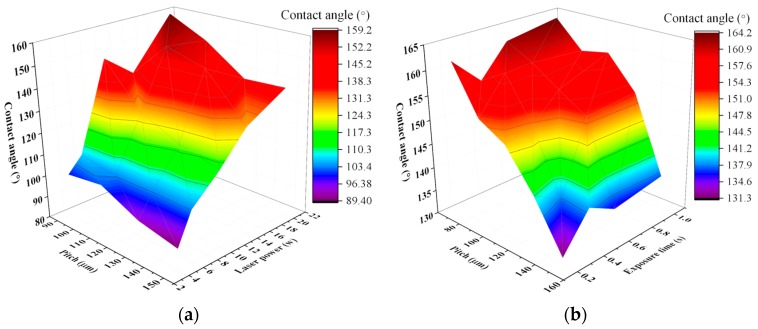
(**a**) 3D colormap of the contact angle as a function of laser power and pitch of microstructures; (**b**) 3D colormap of the contact angle as a function of exposure time and pitch of microstructures.

As shown in Figure 10, 3D colormaps are used to display the relationship between laser power, exposure time, pitch of structures and contact angle. To sum up, the greater contact angle benefit from larger laser power and smaller pitch of microstructures except for some outliers.

**Figure 11:**
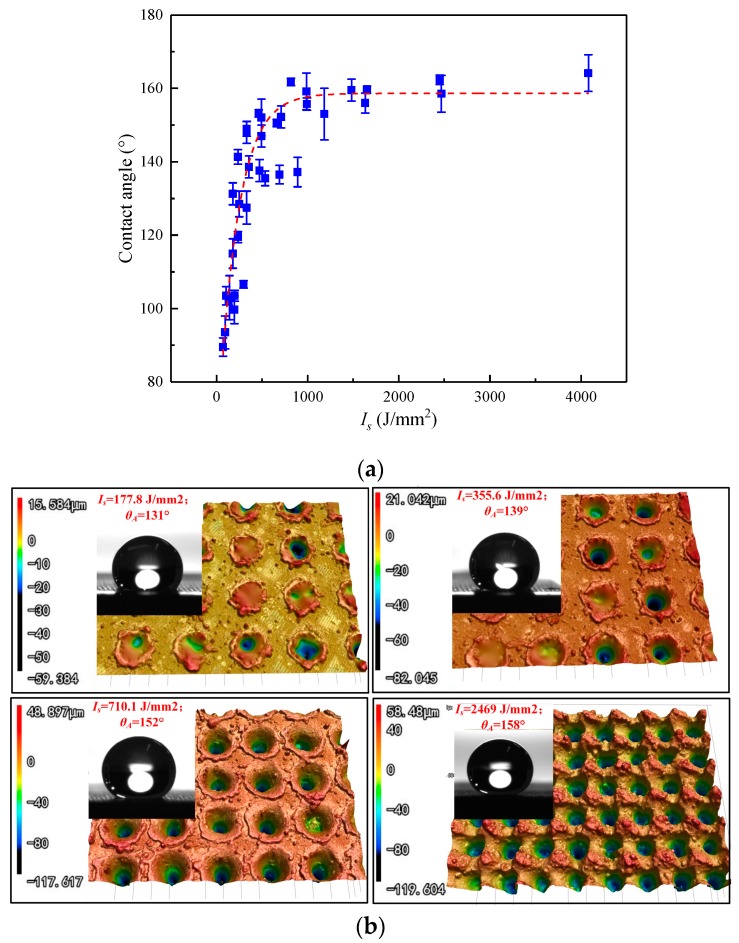
(**a**) Scatter plot and fitted curve between contact angle and *I_s_*; (**b**) Surface morphology and shape of water drops on specimens with a different value of *I_s_*.

Figure 11a shows the scatter diagram and fitted curve between contact angle and *I_s_*. The increasing *I_s_* results in a rapid increase of contact angle at first, and then level off to become asymptotic to the upper limit when *I_s_* greater than 1000 J/mm^2^. The empirical correlation between contact angle and *I_s_* can be expressed by Equation (12). When the value of R_hy_ equals to the threshold value of 0.41, the corresponding *I_s_* is 516.6 J/mm^2^, which is very close to the value of 536 J/mm^2^ obtain from Equation (11). Therefore, *I_s_* should be larger than 536 J/mm^2^ in the laser ablation process, which help ensure the contact angle larger than 150°.

(12) $$\theta_{A}=a-b*e^{d*I_{s}}$$

where, $\theta_{A}$ is contact angle, *a* = 164, *b* = 105, *d* = −0.0039. Coefficients of *a* and *b* have the same meaning with Equation (10).

The surface morphology and shape of water drops on specimens with a different value of *I_s_* are shown in Figure 11b. With the increase of *I_s_*, the depth and density of structures show a significant increasing trend. Thus, the surface topography and contact angle can be well controlled by choosing the appropriate process parameter *I_s_*.

## 5. Conclusions

In this study, the concepts of product and process fingerprint are put forward for the first time to reveal the correlations among process parameters, surface topography and functional performance, i.e., the contact angle of laser ablated superhydrophobic surface on 316L stainless steel. The most appropriate product fingerprint was determined by the indicators of Spearman and Kendall rank correlation coefficients. Then, the candidate that was most sensitive to product fingerprint was determined as the best process fingerprint. Lastly, the correlation between process fingerprint and functional performance was developed. The conclusions can be drawn as follows:

1. The dimensionless surface functional characterization parameter R_hy_, i.e., the average ratio of Rz to Rsm is the most sensitive parameter to contact angle of the specimen, which can be regarded as the product fingerprint.

2. Laser pulse energy per unit area on specimen (*I_s_*) represents the combining effect of laser power, exposure time and pitch of structure on surface topography. It is the best process fingerprint that can be used to control the product fingerprint R_hy_.

3. The increasing *I_s_* leads to the value of R_hy_ increase rapidly at first, and then level off to become asymptotic to the upper limit. A similar trend also can be found between *I_s_* - contact angle and R_hy_ - contact angle. The threshold value of R_hy_ and *I_s_* are 0.41 and 536 J/mm^2^ respectively, which help to ensure the superhydrophobicity (contact angle larger than 150°) of the specimen in the laser ablation process.

## Figures and Tables

**Table 1:** Product fingerprint candidates.

Name	Symbol	Meaning
Arithmetical mean height	Sa	The difference in height of each point compared to the arithmetical mean of the surface.
Maximum height	Sz	The sum of the largest peak height value and the largest pit depth value within the defined area.
Kurtosis	Sku	A measure of the sharpness of the roughness profile. Sku < 3: Height distribution is skewed above the mean plane. Sku = 3: Height distribution is normal. (Sharp portions and indented portions co-exist.) Sku > 3: Height distribution is spiked.
Developed interfacial area ratio	Sdr	The percentage of the definition area’s additional surface area contributed by the texture as compared to the planar definition area.
Root mean square gradient	Sdq	Root mean square of slopes at all points in the definition area. When a surface has any slope, its Sdq value becomes larger.
Average ratio of Rz to Rsm	R_hy_	Average ratio of the maximum height of profile (Rz) and mean width of the profile elements (RSm)

**Table 2:** The laser machining parameters with varied laser power and pitch.

Pitch (μm)	Laser Power (W)	Pulse Repetition Rate	Feed Rate (mm/min)	Exposure Time (s)	Pattern Types
90	4,6,10,14,20	100K	200	0.4	Gaussian holes
110	4,6,10,14,20	100K	200	0.4	Gaussian holes
130	4,6,10,14,20	100K	200	0.4	Gaussian holes
150	4,6,10,14,20	100K	200	0.4	Gaussian holes

**Table 3:** The laser machining parameters with varied exposure time and pitch.

Pitch (μm)	Laser Power (W)	Pulse Repetition Rate	Feed Rate (mm/min)	Exposure Time (s)	Pattern Types
70	20	100K	200	0.2,0.4,0.6,1	Gaussian holes
90	20	100K	200	0.2,0.4,0.6,1	Gaussian holes
110	20	100K	200	0.2,0.4,0.6,1	Gaussian holes
130	20	100K	200	0.2,0.4,0.6,1	Gaussian holes
150	20	100K	200	0.2,0.4,0.6,1	Gaussian holes

**Table 4:** Interpretation of the strength of the correlation coefficient.

Value of Coefficient	Correlation Type
1	Perfect correlation
0.81–0.99	Strong correlation
0.71–0.80	Good correlation
0.51–0.70	Weak correlation
0.01–0.50	Poor correlation
0	No correlation

## Data Statement

All data underpinning this publication are openly available from the University of Strathclyde Knowledge Base at https://doi.org/10.15129/06ca19bc-e028-42b7-ad12-0279d57cc940.
